# ABCA1 promote tumor environment heterogeneity via epithelial mesenchymal transition in Huh7 and HepG2 liver cancer cell

**DOI:** 10.3389/fphar.2024.1498528

**Published:** 2024-12-19

**Authors:** Dinglai Yu, Fang Guo, Qiyu Zhang, Huajun Yu, Wenmin Wang, Yunzhi Chen

**Affiliations:** ^1^ Department of Hepatobiliary Pancreatic Surgery, First Affiliated Hospital of Wenzhou Medical University, Wenzhou, China; ^2^ Department of Gynecology, Wenzhou People’s Hospital, Wenzhou, China; ^3^ The Yangtze River Delta Biological Medicine Research and Development Center of Zhejiang Province (Yangtze Delta Region Institution of Tsinghua University), Hangzhou, Zhejiang, China

**Keywords:** hepatocellular carcinoma, single-cell sequencing, tumor microenvironment, risk stratification, immune therapeutic pathway

## Abstract

In this study, we delve into the intrinsic mechanisms of cell communication in hepatocellular carcinoma (HCC). Initially, employing single-cell sequencing, we analyze multiple malignant cell subpopulations and cancer-associated fibroblast (CAF) subpopulations, revealing their interplay through receptor-ligand interactions, with a particular focus on SPP1. Subsequently, employing unsupervised clustering analysis, we delineate two clusters, C1 and C2, and compare their infiltration characteristics using various tools and metrics, uncovering heightened cytotoxicity and overall invasion abundance in C1. Furthermore, our gene risk scoring model indicates heightened activity of the immune therapeutic pathway in C1. Lastly, employing a formulated scoring system, we stratify patients into high and low-risk groups, revealing notably poorer outcomes in the high-risk cohort on Kaplan-Meier curves. Risk scores exhibit a negative correlation with model genes and immune cell infiltration scores, indicating poor prognosis in the high-risk group. Further characterization elucidates the regulatory landscape of the high and low-risk groups across various signaling pathways. In addition, we used wet lab experiments to prove that ABCA1 plays a pro-oncogenic role in hepatocellular carcinoma cells by promoting proliferation, invasion, migration, and reducing apoptosis. In summary, these findings provide crucial insights, offering valuable clues and references for understanding HCC pathogenesis and patient prognosis.

## 1 Introduction

While hepatocellular carcinoma (HCC) ranks as the fifth most common malignancy globally, it stands as the second leading cause of cancer-related mortality worldwide (Chen K. et al., 2023). In 2020, there were approximately 906,000 new cases and 830,000 deaths attributed to HCC, with an incidence of 4.7% and a mortality rate of 8.3% (Sung et al., 2021). In China, there were an estimated 431,383 new cases and 412,216 deaths from HCC in 2022, representing roughly half of the global increase in HCC cases and deaths (Fu et al., 2023). The incidence of HCC is rapidly increasing among both males and females (Islami et al., 2017; Jeong et al., 2018), notably serving as a primary cause of cancer-related mortality in transitional countries such as Mongolia, Thailand, Cambodia, Egypt, and Guatemala (Sung et al., 2021; Tong et al., 2020). HCC constitutes 80%–90% of primary liver cancers, with cholangiocarcinoma (CCA) accounting for 10%–15%, while vascular sarcomas and hepatoblastomas represent a smaller proportion (Li et al., 2021). Chronic inflammatory etiologies, including hepatitis B virus (HBV), hepatitis C virus (HCV) infections, alcoholic steatohepatitis (ASH), non-alcoholic steatohepatitis (NASH), aflatoxin exposure, cirrhosis, smoking, obesity, diabetes, iron overload, various dietary habits, and sedentary lifestyle, are major risk factors for HCC (Li et al., 2021; Anwanwan et al., 2020; Li and Wang, 2016; Duan et al., 2014). HCC may present without evident signs or symptoms, with nonspecific manifestations including right upper quadrant pain, abdominal distension, jaundice, poor appetite, persistent fatigue, and weight loss (Mokdad et al., 2015). Histologically, HCCs are classified by the World Health Organization (WHO) into well-differentiated, moderately differentiated, poorly differentiated, and undifferentiated subtypes, with growth patterns including capsule invasion, infiltration into adjacent liver parenchyma, satellite nodule formation, tumor thrombus formation, and intrahepatic metastasis (Li and Wang, 2016). The incidence of metastatic liver cancer is 18–40 times higher than that of primary hepatic malignancies, owing to the unique anatomical microenvironment of the liver facilitating colonization by extrahepatic cancer cells (including colorectal, pancreatic, breast, melanoma, and lung cancers) (Liu et al., 2023). Liver metastasis significantly impacts both the 5-year survival rate and quality of life (Li et al., 2021), with only approximately 20% of patients with extrahepatic metastases being suitable for surgery (Zhou et al., 2016). Early-stage HCC may benefit from partial hepatectomy, ablation therapy, or liver transplantation, with varying prognostic outcomes. However, the local failure rate of ablation therapy is significantly higher than that of surgical resection, and percutaneous ablation in the pre-transplant setting carries a risk of tumor dissemination, potentially rendering initially transplant-eligible patients ineligible (Bruix et al., 2015). Liver transplantation is limited by donor scarcity and delays between transplant indications and surgery (Soulen and García-Mónaco, 2021), with a median 5-year survival rate of approximately 70%. Nevertheless, 15% of liver transplant recipients experience recurrence post-treatment, with a median 5-year survival rate ranging from 20% to 35%, complicated by the anatomical challenges of early cancer detection (Li and Wang, 2016; Gao et al., 2021; Cheng et al., 2016). For nearly half of HCC patients diagnosed in advanced stages, conventional treatments such as curative resection and ablation therapy may be precluded, although options such as targeted drug therapy or immunotherapy remain available (Zhou et al., 2016; Chen Y. et al., 2023). Sorafenib, an orally administered kinase inhibitor targeting tumor cells, represents a relatively novel therapeutic option for HCC patients with advanced or metastatic disease. However, fewer than one-third of eligible patients benefit from sorafenib, with associated adverse events and a median time to resistance of less than 6 months from initiation of treatment (Anwanwan et al., 2020; Hao et al., 2023). Therefore, comprehensive research into the mechanisms underlying HCC development and progression is imperative, particularly for identifying more effective treatment modalities and elucidating the role of key genetic factors, which are crucial for the diagnosis, treatment, and prognosis of HCC.

As the culmination of our introduction, this study aims to unravel the intricate mechanisms of cell communication within HCC. Utilizing single-cell sequencing, we dissect the interplay between multiple malignant cell subpopulations and cancer-associated fibroblasts (CAFs), with a special emphasis on SPP1-mediated receptor-ligand interactions. Through unsupervised clustering, we identify two distinct clusters, C1 and C2, and characterize their infiltration patterns, revealing elevated cytotoxicity and invasion in C1. Our gene risk scoring model further highlights heightened immune therapeutic pathway activity in C1. Moreover, patient stratification based on a formulated scoring system demonstrates poorer outcomes in the high-risk group. Wet lab experiments validate the oncogenic role of ABCA1 in promoting HCC cell proliferation, invasion, migration, and reducing apoptosis. Collectively, our findings offer novel insights into HCC pathogenesis and patient prognosis, laying the groundwork for future research and therapeutic strategies.

## 2 Material and methods

### 2.1 Data collection and preprocessing

Firstly, we retrieved bulk transcriptomic data and corresponding clinical information for HCC from The Cancer Genome Atlas (TCGA, https://portal.gdc.cancer.gov/) database. Additionally, we obtained two bulk RNA-seq datasets, GSE14520 and GSE76427, from The Gene Expression Omnibus (GEO, https://www.ncbi.nlm.nih.gov/geo/) database. Furthermore, we downloaded the ICGC-JP dataset from the International Cancer Genome Consortium (ICGC, https://dcc.icgc.org/) database. Finally, three single-cell sequencing datasets for HCC, namely, GSE146115, GSE146409, and GSE166635, were obtained from TISCH2 (http://tisch.comp-genomics.org/home/) database. All publicly available databases utilized in this study permit unrestricted access and utilization without additional ethical approval. Our data retrieval and analysis procedures adhere to relevant guidelines. We standardized all sequencing data into Transcripts per million (TPM) format. Records with missing information were excluded, and in cases where a gene had multiple entries, the mean value was calculated across all entries.

### 2.2 Single-cell sequencing data analysis

Utilizing the “Seurat” package and the SCP pipeline (https://github.com/zhanghao-njmu/SCP), we conducted analysis on the single-cell sequencing data. To ensure the accuracy and reliability of subsequent research, we initially performed quality control on the acquired data. Our criteria were as follows: percent. mt <25, nFeature_RNA <9,000. Additionally, we employed the “harmony” package to integrate and batch-correct the quality-controlled single-cell data. Subsequently, we employed Uniform Manifold Approximation and Projection (UMAP) for dimensionality reduction and clustering of the single-cell data. We annotated and visualized several major cell types based on relevant information provided by the TISCH database. Concurrent with cell annotation, we validated the subclasses by cross-referencing the gene expression profiles with established cell type annotations.

To investigate the interaction and communication between malignant cell clusters and Cancer-Associated Fibroblasts (CAFs) clusters, we performed UMAP dimensionality reduction again for both cell types based on the EPCAM expression levels of malignant cell clusters (n = 7,186) and the expression levels of COL1A1 and COL1A2 for CAFs clusters (n = 698). We further subdivided them into several cell subclusters and visualized the results. Next, we utilized RunSlingshot to construct developmental trajectories of malignant and CAFs cell subclusters and predicted their developmental paths.

Subsequently, we conducted Differentially Expressed Genes (DEGs) analysis for each cell subcluster, with parameters set as follows: fc.threshold = 1, only.pos = FALSE. Finally, we performed Gene Ontology Biological Process (GO_BP) enrichment analysis for each cell subcluster and selected the top six statistically significant GO_BP enrichment terms for visualization. Parameters were set as follows: db = “GO_BP,” species = “Homo_sapiens,” DE_threshold = “avg_log_2_FC > log_2_ (1.5) & p_val_adj <0.05.”

### 2.3 Analysis of cell communication

We conducted an analysis of cell communication by using the CellChat and NicheNet algorithms on various cell subpopulations. Firstly, we presented an interaction network in the form of a chord diagram, demonstrating the frequency and strength of interactions among different subpopulations of malignant and CAFs cells. Subsequently, we visualized the ligand-receptor relationships and pairings between different cell pathways within each subpopulation. Additionally, we focused on the expression patterns of SPP1 as a ligand and its various receptors in the malignant and CAFs subpopulations, using a violin plot. Furthermore, we analyzed the significance of different subpopulations in the SPP1 signaling pathway. To explore the interaction network within each cell subpopulation, we created scatter plots to display the outward and inward interaction strengths of each subpopulation. Finally, by using certain genes in the CAFs subpopulation as ligands and genes in the malignant subpopulation as receptors, we analyzed the binding potential and biological effects of these ligand-receptor interactions, which were visualized using a heat map.

### 2.4 Constructing gene regulatory networks

We utilized the “SCENIC” R package to construct GRNs for HCC. Leveraging the single-cell dataset of HCC and relevant algorithms, we particularly focused on the distribution and expression patterns of five regulatory factors associated with HCC (BRF1_extended_29g, ARNTL_extended_39g, ARNTL_24g, BCLAF1_extended_22g, ATF3_extended_16g) across various cell subpopulations, visualized using UMAP. Additionally, we generated a heatmap illustrating the differential activity levels of these five regulatory factors between malignant and CAFs cells. Subsequently, we amalgamated all target genes regulated by these five factors into a signature and proceeded with further analysis based on this signature.

### 2.5 Unsupervised clustering and correlation analysis

Utilizing the aforementioned signature, we conducted unsupervised clustering analysis using the “ConsensusClusterPlus” R package with the following parameters: maxK = 9, reps = 1,000, pItem = 0.8, pFeature = 1, tmyPal = color, title = “ConsensusCluster/,” clusterAlg = “km,” distance = “euclidean,” seed = 123,456. By subjecting tumor tissue samples to hierarchical clustering, we attempted sample grouping. Subsequently, leveraging Cumulative Distribution Function (CDF) curves and Proportion of Ambiguous Clustering (PAC) scores, we selected the most appropriate k value for grouping, resulting in two distinct clusters (C1 and C2). Furthermore, we employed the TCGA-LIHC dataset and conducted log-rank testing to plot Kaplan-Meier (KM) curves, demonstrating survival disparities between the different clusters.

### 2.6 Differential analysis of HCC tumor microenvironment

To gain insight into the disparities within the HCC TME across distinct clusters, we leveraged the clustering results to conduct comparative analyses of the TME in clusters C1 and C2. Initially, employing Single-sample Gene Set Enrichment Analysis (ssGSEA), we evaluated the relative infiltration abundance of diverse immune cell subtypes within the two clusters. Subsequently, we depicted the disparities in the activity levels of CYT (cytotoxic activity), GFP (T cell inflamed gene expression profile), IFNG (INF-γ), and TMB (tumor mutation burden) between the two clusters using box plots. Additionally, we assessed the infiltration abundance of immune cell subtypes in both clusters using five TME deconvolution algorithms (CIBERSORT, MCP-counter, quanTIseq, EPIC, and TIMER) from the “IOBR” R package (https://github.com/IOBR/IOBR), scoring the results accordingly. Furthermore, we downloaded 150 immunomodulators and chemokines from the TISIDB database (http://cis.hku.hk/TISIDB/), including 41 chemokines, 24 immunoinhibitors, 46 immunostimulators, 21 Major Histocompatibility Complex (MHC), and 18 receptors. Based on this data, we constructed a heatmap illustrating the expression profiles of relevant immune regulatory molecules across different clusters. Finally, employing Gene Set Variation Analysis (GSVA), we enriched scores for the anti-cancer immunity cycle and immunotherapy-predicted pathways in the two clusters, followed by an analysis of the disparities between the clusters.

### 2.7 Gene set enrichment analysis

Initially, we utilized the “limma” package to identify differentially expressed genes between clusters C1 and C2. Subsequently, employing the “clusterprofiler” R package, we conducted GSEA to delineate the signaling pathways enriched and discovered the upregulated cancer signatures within both clusters. Concurrently, data visualization was performed using the “GseaVis” R package to generate bubble plots illustrating the results. Additionally, GSEA was employed to identify both upregulated and downregulated signaling pathways within the C1 cluster.

### 2.8 Construction of prognostic models

Based on the communication signature between malignant and CAFs cellular subgroups, we employed the Least Absolute Shrinkage and Selection Operator (LASSO) method to screen prognostic marker genes within the TCGA-LIHC dataset. Subsequently, utilizing the multiCOX analysis approach, we constructed a prognostic model for HCC. Employing the model formula, each patient was assigned a score, yielding a RiskScore for every sample. The RiskScore is defined by summing the product of gene expression levels and their corresponding coefficients, as demonstrated below:

$$\left.Risk\;score=\sum_{i=1}^{n}\left[{\mathrm{Exp}}_{{gene}_{i}}*\beta_{i}\right.\right]$$

Here, ${\mathrm{Exp}}_{{gene}_{i}}$ represents the expression level of the model gene, and $\beta_{i}$ represents the corresponding coefficient of the model gene. Additionally, we visualized the coefficients of the prognostic model through a lollipop plot of feature gene coefficients. Based on the median score, patients were divided into high-risk and low-risk groups. Using the TCGA dataset (n = 329), we plotted KM curves to analyze the prognosis of the two risk groups and constructed Receiver Operating Characteristic (ROC) curves to analyze the model’s performance at 1, 3, and 5 years. We define a model as having good diagnostic performance in this dataset when the area under the curve (AUC) exceeds 0.6. Subsequently, we validated the prognostic model in external validation sets GSE76427 (n = 115), GSE14520 (n = 242), and ICGC-JP (n = 240). We utilized KM curves and ROC curves to validate the predictive ability of the model in different datasets. Next, we conducted correlation analysis, demonstrating the correlation between RiskScore and various immune checkpoint levels and immune cell infiltration levels through a correlation heatmap. Using the “limma” package, we performed DEGs analysis between the high-risk and low-risk groups, identifying differentially expressed genes between the two groups. Finally, through GSEA, we analyzed the abnormal signaling pathways that were upregulated and downregulated in the high-risk group.

### 2.9 Cell culture and transfection

We used human liver cancer cell lines HepG2 and Huh7 (Cell Bank of the Chinese Academy of Sciences). Huh7 cells were cultured in DMEM (HyClone, United States), and HepG2 cells in MEM (HyClone, United States), both supplemented with 10% FBS (BI, Israel) and 100 U/mL penicillin/100 μg/mL streptomycin (HyClone, United States). Cells were maintained in a humidified CO2 incubator at 37°C.

For transfection, Huh7 cells were treated with ABCA1 shRNA (Sangon, China) to knock down expression, while HepG2 cells were transfected with an ABCA1 overexpression plasmid (with a negative control). Cells were resuspended in complete medium and seeded into 6-well plates at 1 × 10^4^ cells/well with 2 mL of medium. Transfection was performed using PolyFast reagent (MCE, United States, catalog number HY-K1014) according to the manufacturer’s instructions. After a 15-min incubation at room temperature, the cells were re-incubated. The medium was refreshed 6 h post-transfection, and subsequent experiments were conducted 48 h later.

### 2.10 RT-qPCR and total RNA extraction

We used RT-qPCR to measure ABCA1 mRNA expression in different cell groups. Cells in 6-well plates were trypsinized (KeyGEN, China), washed with PBS, and centrifuged at 4°C (800–2,000 rpm). RNA was extracted using 800–1,000 μL Trizol (Takara, Japan), followed by chloroform precipitation and ethanol/isopropanol purification (SINOPHARM, China). The RNA was resuspended in 20 μL DEPC-treated water and quantified using a Nanodrop 2000 spectrophotometer (Thermo, United States). Reverse transcription was performed with the PrimeScript RT reagent kit (TaKaRa, Japan), and RT-qPCR was conducted using SYBR GreenER Supermix (TaKaRa, Japan) on a 7,500 Real-Time PCR System (Thermo Fisher Scientific, United States) according to the manufacturer’s protocols. ABCA1 expression was quantified using the 2^−ΔΔCT^ method, normalized to β-actin.

### 2.11 Colony formation assay

Colony Formation Assay was employed to determine differences in colony numbers among different cell lines. Cells were initially seeded at a density of 1 × 10^3^ cells per well in a 6-well plate, gently agitated, and subsequently cultured in a cell culture incubator for approximately 14 days. Following removal of the culture medium, the cells underwent three washes with PBS. Colonies underwent fixation using formaldehyde for 15 min, followed by staining with 1 mL of 0.5% crystal violet (Solarbio, China), three subsequent PBS washes, air-drying, and subsequent imaging and quantification.

### 2.12 CCK8 assay

After 48 h post-transfection, Huh7 and HepG2 cell lines were plated into 96-well plates at a density of 6,000 cells per well and returned to the incubator for adherence. Each experimental group was replicated three times. The CCK-8 reagent (KeyGEN, China) was reconstituted as per the manufacturer’s instructions by diluting it with complete culture medium to achieve a final volume of 200 μL per well. Using a pipette, the prepared solution was swiftly aliquoted into the wells of the 96-well plates. The plates were shielded from light exposure by covering them with aluminum foil, and absorbance readings at 450 nm were taken using a spectrophotometer following a 2-h incubation period. Subsequent measurements were taken at 24, 48, 72, and 96-h time points, repeating the aforementioned steps.

### 2.13 EDU assay

We used the EdU assay to assess proliferation level differences among different groups of Huh7 and HepG2 cells. Following a 48-h transfection period, the culture medium was removed, and cells were washed three times with PBS. As per the protocol, cells were permeabilized with 0.3% Triton X-100 (Beyotime, China) for 25 min at room temperature. After permeabilization, cells were incubated with EdU reaction mixture to allow EdU incorporation into newly synthesized DNA. Subsequently, cells were washed again with PBS and fixed with a fixing solution. Following fixation, cells were stained with a fluorescent azide to visualize EdU incorporation. After washing to remove excess stain, cells were counterstained with DAPI for 10 min to visualize nuclei. Finally, each well was washed with PBS, and anti-fluorescence quenching reagent (Beyotime, China) was added to preserve the fluorescence signal. The plates were then examined, and images were captured using a fluorescent microscope.

### 2.14 Wound healing assay

Following 48 h of transfection, the medium was aspirated, and PBS was introduced. Using a precise ruler for guidance, a deliberate single straight scratch was introduced into each well using a 200 μL pipette. The pipette tip was substituted after each well, and cells underwent three PBS washes. Subsequently, each well received basic culture medium lacking FBS. At this point, microscopic images were captured to document the initial scratch, measure the wound area, and define this moment as time point zero. After incubating the cells in a cell culture incubator for 48 h, images were taken again to measure the healed wound area and calculate the percentage of scratch closure.

### 2.15 Total protein extraction and Western blot analysis

Western blotting was used to assess protein expression of ZO-1, E-cadherin, Vimentin, Slug, ABCA1, and β-actin in Huh7 and HepG2 cells. Cells were lysed using RIPA buffer with protease inhibitors (100:1), sonicated (40% amplitude, 1s pulses, 3 cycles), and incubated on ice for 30 min with shaking. Lysates were centrifuged (10,000 rpm, 15 min, 4°C), and supernatants were collected for protein quantification. Samples were prepared with loading buffer, heated (95°C, 5 min), and subjected to electrophoresis (20 μg/lane, 10% SDS-PAGE, 100V). Proteins were transferred to a PVDF membrane (0.45 μm), blocked (10 min), and incubated with primary antibodies overnight at low temperature. After washing, membranes were incubated with HRP-conjugated secondary antibodies (1.5 h, RT) and visualized using an ECL kit. Antibodies were sourced from Proteintech.

### 2.16 Transwell assay

Transwell chambers (Thermo, United States) were coated with extracellular matrix gel (1:8 dilution, 40 μL/chamber) and dried for 24 h. Cells (20,000/chamber) were seeded in serum-free medium (200 μL/chamber) on a 24-well plate with 500 μL complete medium per well. After 20-h incubation in a CO2 incubator, non-invading cells were removed, and chambers were fixed with 4% paraformaldehyde, washed, and stained with 0.1% crystal violet. Microscopic images were then captured.

### 2.17 Flow cytometry for detecting cell apoptosis

Flow cytometry was employed to assess apoptosis in Huh7 and HepG2 cells. Following reagent centrifugation, cells were washed, digested with trypsin (without EDTA, with 3-min interval checks), and centrifuged at 2,000 rpm for 5 min. After two additional PBS washes, cells were suspended in 400 μL of binding buffer. Annexin V FITC/PI staining solution was added, followed by a 15-min incubation at 37°C. Cells were then transferred to flow cytometry tubes and filtered through a nylon mesh. The FL1 channel (for FITC green fluorescence) and FL3 channel (for PI red fluorescence, Ex = 488 nm, Em ≥ 630 nm) were used for analysis. Voltage and compensation settings on the flow cytometer were adjusted to ensure that 99% of cells occupied the lower left quadrant.

### 2.18 Statistical analysis

All statistical analyses were conducted using R software (version 4.1.3). Differential gene expression analysis was performed using the “limma” package. The “ggplot2” package was employed as the primary tool for visualization. A threshold of *p* < 0.05 was considered statistically significant (*p < 0.05; **p < 0.01; ***p < 0.001; ****p < 0.0001).

## 3 Results

### 3.1 Single-cell data analysis of malignant cell populations

**FIGURE 1:**
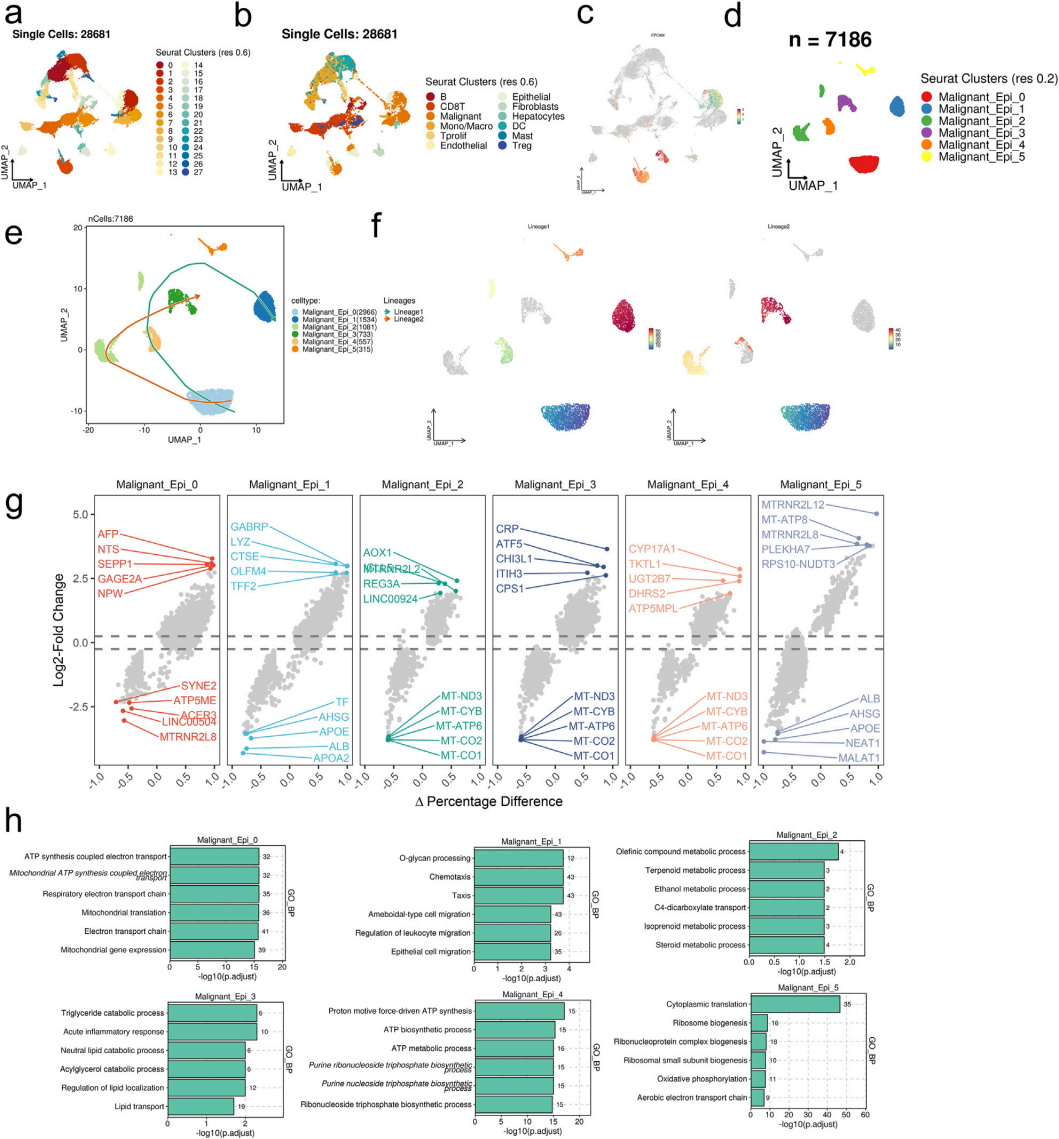
scRNA-seq analysis unravels the heterogeneity of in malignant cells in HCC. **(A)** 28 clusters were identified in the integrated scRNA-seq dataset. **(B)** 12 major cell types were annotated. **(C)** UMAP visualization of the expression levels of EPCAM in the integrated scRNA-seq dataset. **(D)** Malignant cell subpopulations were identified from the major malignant cell set. **(E, F)** The predicted developmental trajectories of malignant epithelial cell subsets. **(G)** The differentially expressed genes of each malignant cell subset. **(H)** Top six enriched GO_BP terms of each malignant cell subset.

Utilizing integrated single-cell sequencing data, UMAP dimensionality reduction clustering identified 28 clusters, subsequently annotated into 12 major cell types based on information provided by the TISCH database (Figures 1A, B). Further analysis of the target malignant cell population delineated it into 6 cellular subgroups: Malignant_Epi_0, Malignant_Epi_1, Malignant_Epi_2, Malignant_Epi_3, Malignant_Epi_4, and Malignant_Epi_5 (Figures 1C, D). Developmental trajectory prediction revealed two trajectories: Lineages1 (Malignant_Epi_0- Malignant_Epi_4- Malignant_Epi_1) and Lineages2 (Malignant_Epi_0- Malignant_Epi_2- Malignant_Epi_3), all originating from Malignant_Epi_0 (Figures 1E, F).

In differential gene expression analysis, genes associated with metabolism, immune inflammation, neuroregulation, and cell signaling pathways were upregulated across the 6 cellular subgroups, while genes related to mitochondria, ribosomes, long non-coding RNA, lipoproteins, and plasma proteins were downregulated (Figure 1G). GO_BP enrichment analysis revealed statistically significant enrichment of the top 6 pathways across the 6 cellular subgroups, including cellular mitochondrial functions, mobility and migration, metabolic processes, lipid metabolism regulation and transport processes, ATP synthesis and metabolism, protein synthesis, and energy metabolism (Figure 1H).

### 3.2 Single-cell data analysis of CAF cell population

**FIGURE 2:**
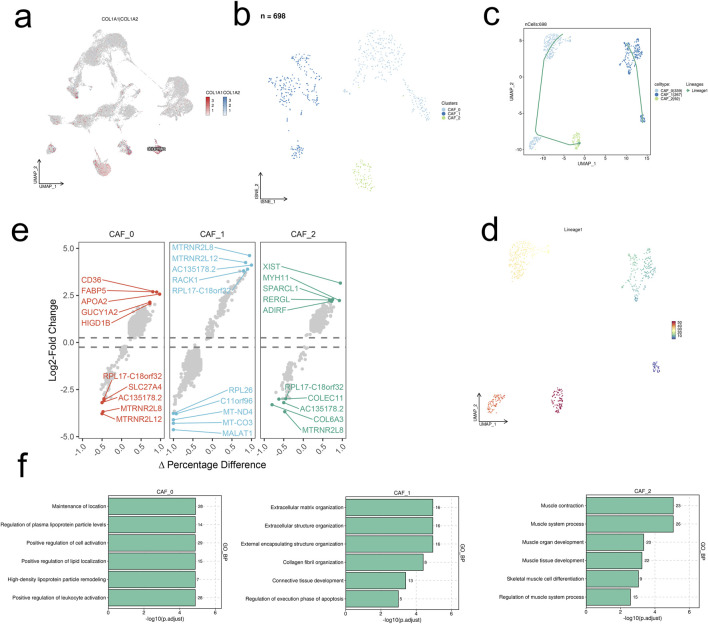
scRNA-seq analysis unravels the heterogeneity of CAFs in HCC. **(A)** UMAP visualization of the expression levels of COL1A1 and COL1A2 in the integrated scRNA-seq dataset. **(B)** UMAP visualization of the 698 CAFs. **(C, D)** The predicted developmental trajectories of CAF subsets. **(E)** The differentially expressed genes of each CAF subset. **(F)** Top six enriched GO_BP terms of each CAF subset.

Furthermore, we conducted a detailed analysis of the CAF cell population, reducing its dimensionality into 3 groups using UMAP: CAF_0, CAF_1, and CAF_2 (Figures 2A, B). Predicted developmental trajectories revealed a single trajectory: Lineages1 (CAF_1, CAF_0, CAF_2) (Figures 2C, D). In the analysis of Differentially Expressed Genes (DEGs), genes related to cell structure and signaling transduction, protein synthesis, and nucleic acid metabolism were found to be upregulated across the 3 cellular subgroups, while genes associated with mitochondria, ribosomes, mitochondrial and nuclear-encoded RNA, extracellular matrix proteins, and receptors were downregulated (Figure 2E). GO_BP enrichment analysis indicated statistically significant enrichment of the top 6 pathways across the 3 cellular subgroups, including maintenance of normal biological functions and homeostasis, extracellular matrix, and the muscular system (Figure 2F).

### 3.3 Cellular communication analysis and construction of gene regulatory networks

**FIGURE 3:**
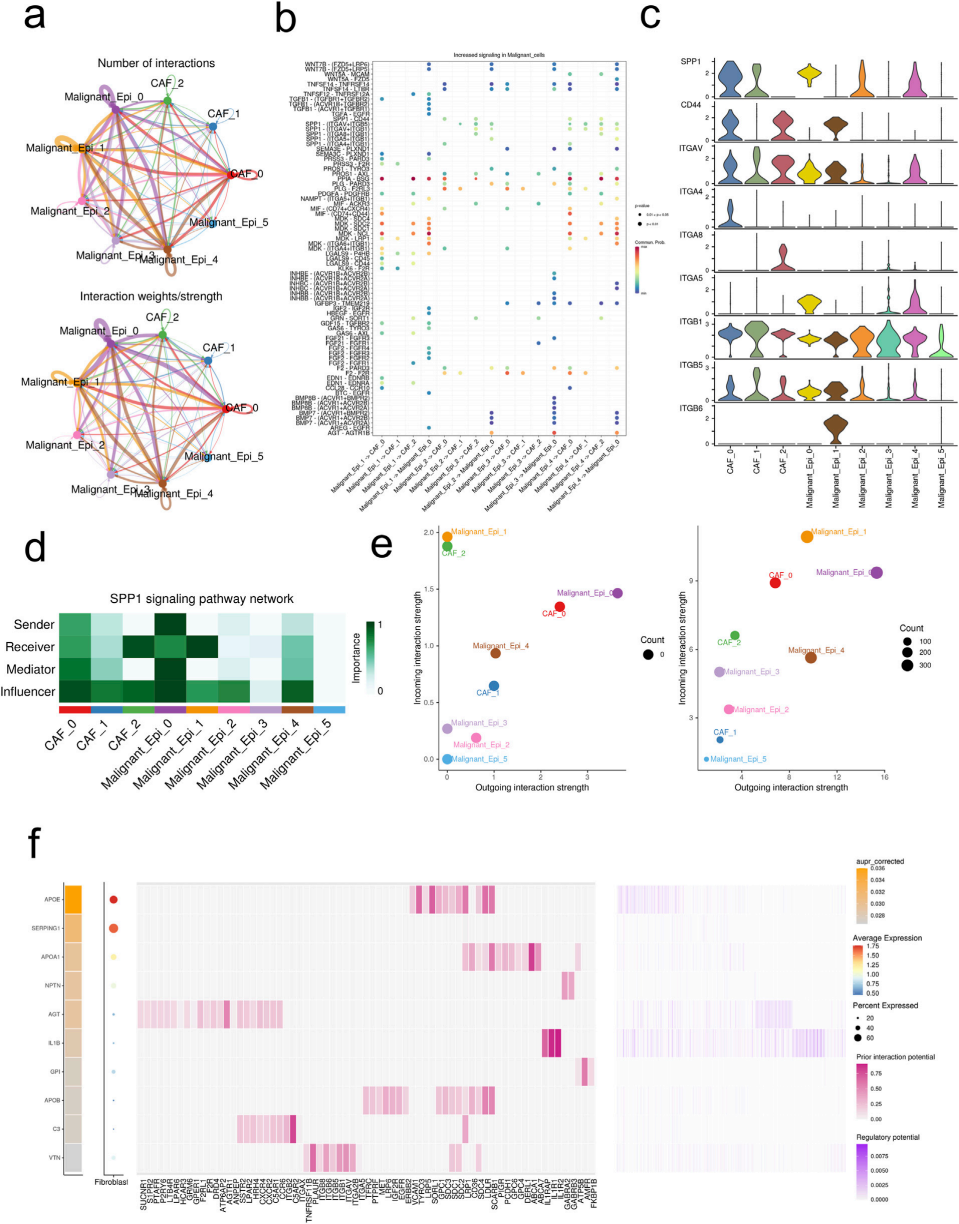
Intercellular communications between CAFs and malignant cells. **(A)** The intercellular interactions between subsets of CAFs and malignant cells. **(B)** The ligand-receptor pairs between CAFs and malignant cells. **(C)** Expression profiles of SPP1 signaling pathway in CAFs and malignant cells. **(D)** The importance of each subset of CAFs and malignant cells in the SPP1 signaling pathway. **(E)** The incoming/outgoing strength of each subset of CAFs and malignant cells in the SPP1 signaling pathway (left) and the whole signaling pathways (right). **(F)** Top ligands in the communication network. Ligand-target gene matrix denoting the potential regulatory relationships between ligands and target genes among CAFs and malignant cells. The color intensity represented the regulatory potentials.

**FIGURE 4:**
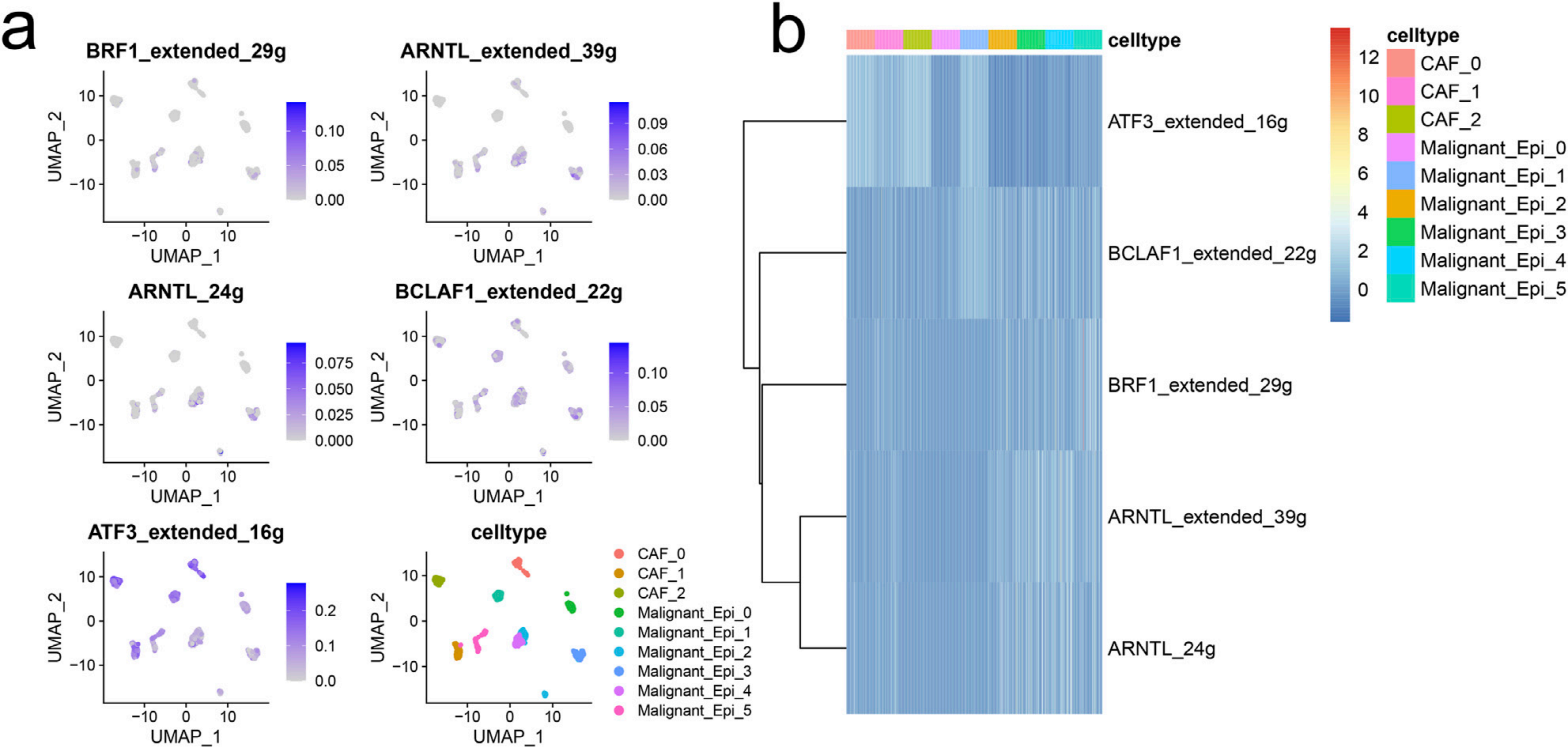
The gene regulatory networks (GRNs) in HCC. **(A)** UMAP visualization of the five regulons at single-cell level of HCC. **(B)** Heatmap demonstrated the activity of each regulon in CAFs and malignant cells.

The figure demonstrates that the interactions and strengths among Malignant_Epi_0, Malignant_Epi_1, Malignant_Epi_4, CAF_0, and other cellular subgroups are relatively strong, while Malignant_Epi_2, Malignant_Epi_3, Malignant_Epi_5, CAF_1, and CAF_2 exhibit weaker interactions (Figure 3A). We investigated the receptor communication relationships within different cellular subgroups, focusing particularly on the SPP1 receptor relationships. SPP1 expression levels are notably higher in CAF_0, CAF_1, Malignant Epi 0, Malignant Epi 2, and Malignant Epi 4 subgroups. Among the potential targets of SPP1, ITGB1 is actively expressed in all cellular subgroups, whereas ITGA4, ITGA8, and ITGB6 are inactive in most cellular subgroups (Figures 3B, C). Analysis of the SPP1 signaling pathway network reveals that Malignant_Epi_0 exhibits higher importance in Sender, Receiver, Mediator, and Influencer aspects, while Malignant_Epi_3 demonstrates lower importance (Figure 3D). Both CAF cellular subgroups and malignant cell populations exhibit weaker outward and inward interaction strengths in the SPP1 pathway compared to the entire signaling pathway (Figure 3E). The ligand-receptor gene matrix indicates binding potential and biological effects only when IL1B serves as the ligand and IL1RAP, IL1R1, IL1R2 serve as receptors (Figure 3F). Additionally, using the “SCENIC” package, we focused on five regulatory factors at the single-cell level in HCC (BRF1 _extended _29g, ARNTL_extended _39g, ARNTL _24g, BCLAF1_extended _22g, ATF3_extended _16g). We found that ATF3_extended _16g is expressed at higher levels in three CAF cellular subgroups and six Malignant cellular subgroups compared to the other four factors (Figure 4A). Heatmap results indicate that, except for ATF3_extended _16g, the remaining regulatory factors exhibit high expression in the Malignant_Epi_5, Malignant_Epi_4, Malignant_Epi_3, and Malignant_Epi_2 cellular subgroups (Figure 4B).

### 3.4 Unsupervised clustering and survival disparity analysis

**FIGURE 5:**
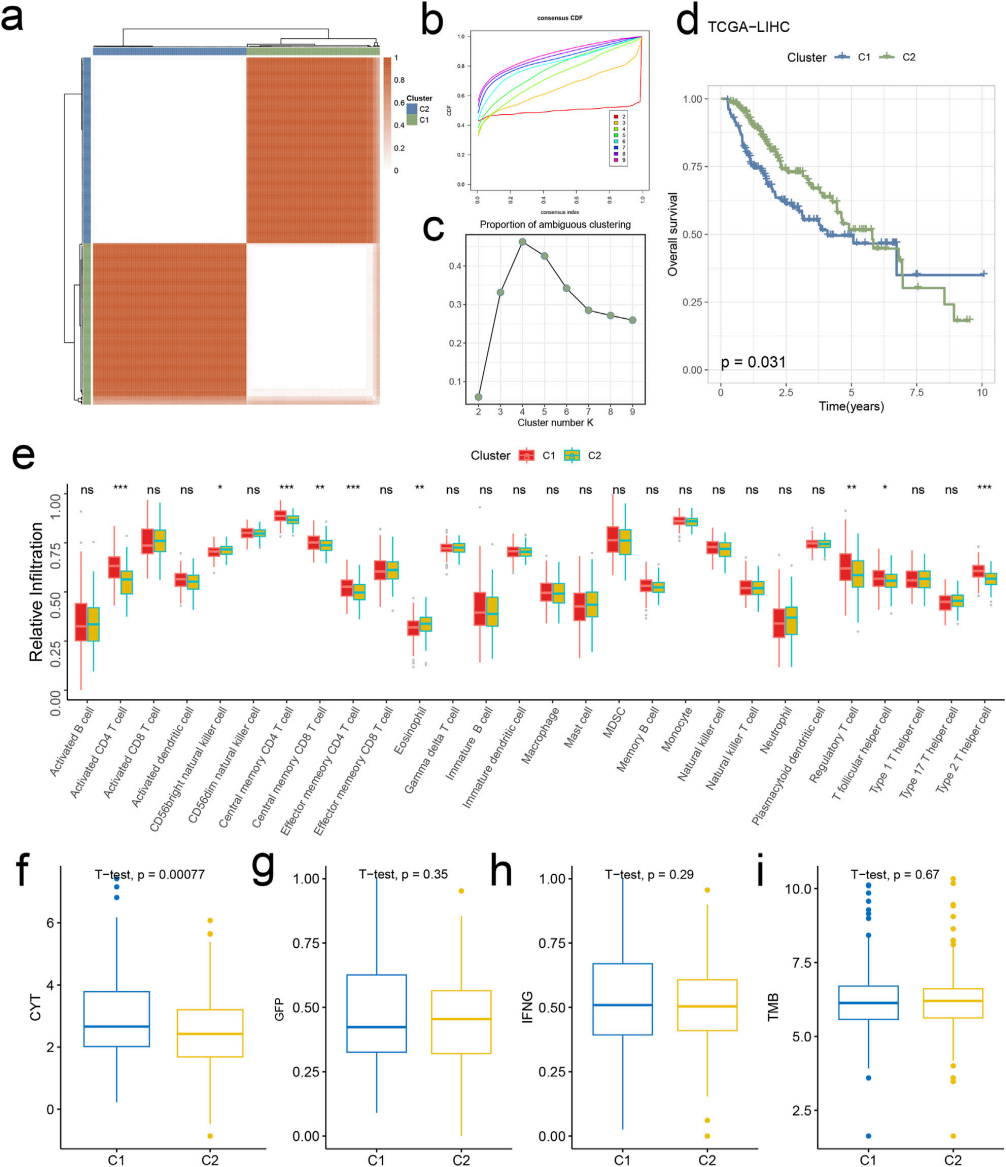
Signature stratifies HCC TME into two subclusters with distinct prognosis and biological features. **(A)** The consensus score matrix of all samples when k = 2. A higher consensus score denotes higher similarity. **(B)** The CDF curves of the consensus matrix for each k (indicated by colors). **(C)** The PAC score for each k. **(D)** KM survival curves with log-rank test demonstrate survival discrepancies between two clusters. **(E)** Relative infiltration abundances of 28 immune cell subsets in two clusters. *p* values are determined by the Wilcoxon test. ns: non-significant; *p < 0.05; ***p < 0.001. The activities of CYT **(F)**, GFP **(G)**, IFNG **(H)**, and TMB **(I)** between two clusters.

In this section, we explore unsupervised clustering of tumor tissue samples and investigate survival disparities. Utilizing hierarchical clustering, we identified k = 2 as the optimal grouping based on CDF curve analysis and PAC scores. Notably, the consensus matrix plot exhibited robust intra-cluster cohesion and inter-cluster distinctiveness (Figures 5A–C). Kaplan-Meier survival curves unveiled significant survival discrepancies between the two clusters, with cluster 1(C1) displaying inferior prognosis (*p* = 0.031, Figure 5D).

### 3.5 Analysis of HCC tumor microenvironment disparities

**FIGURE 6:**
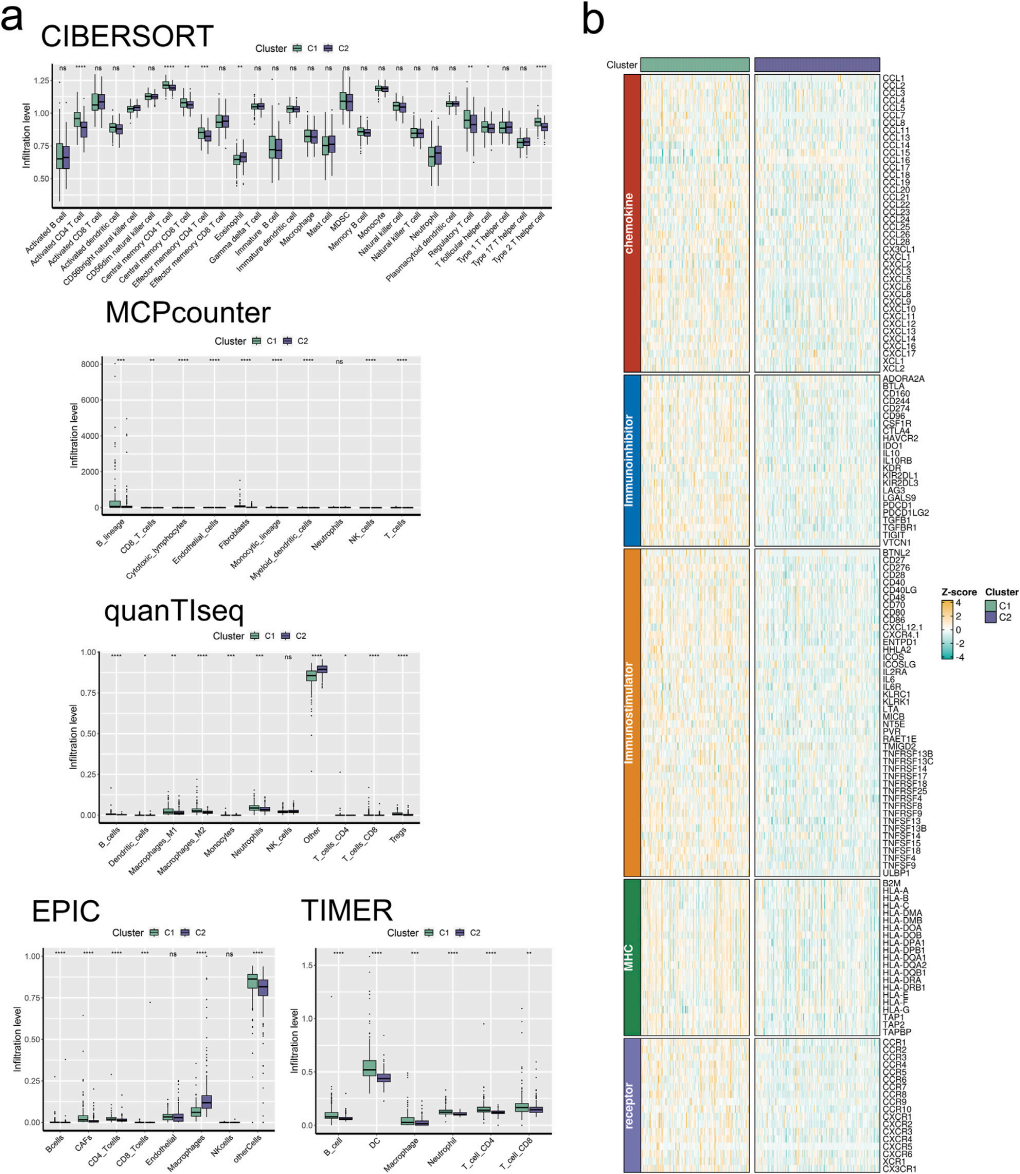
Signature stratifies HCC TME into two subclusters with distinct TME landscapes. **(A)** The infiltration abundance of immune cell subsets evaluated by CIBERSORT, MCP-counter, quanTIseq, EPIC, and TIMER for two clusters. **(B)** The expression abundances of immunoregulators for two clusters.

**FIGURE 7:**
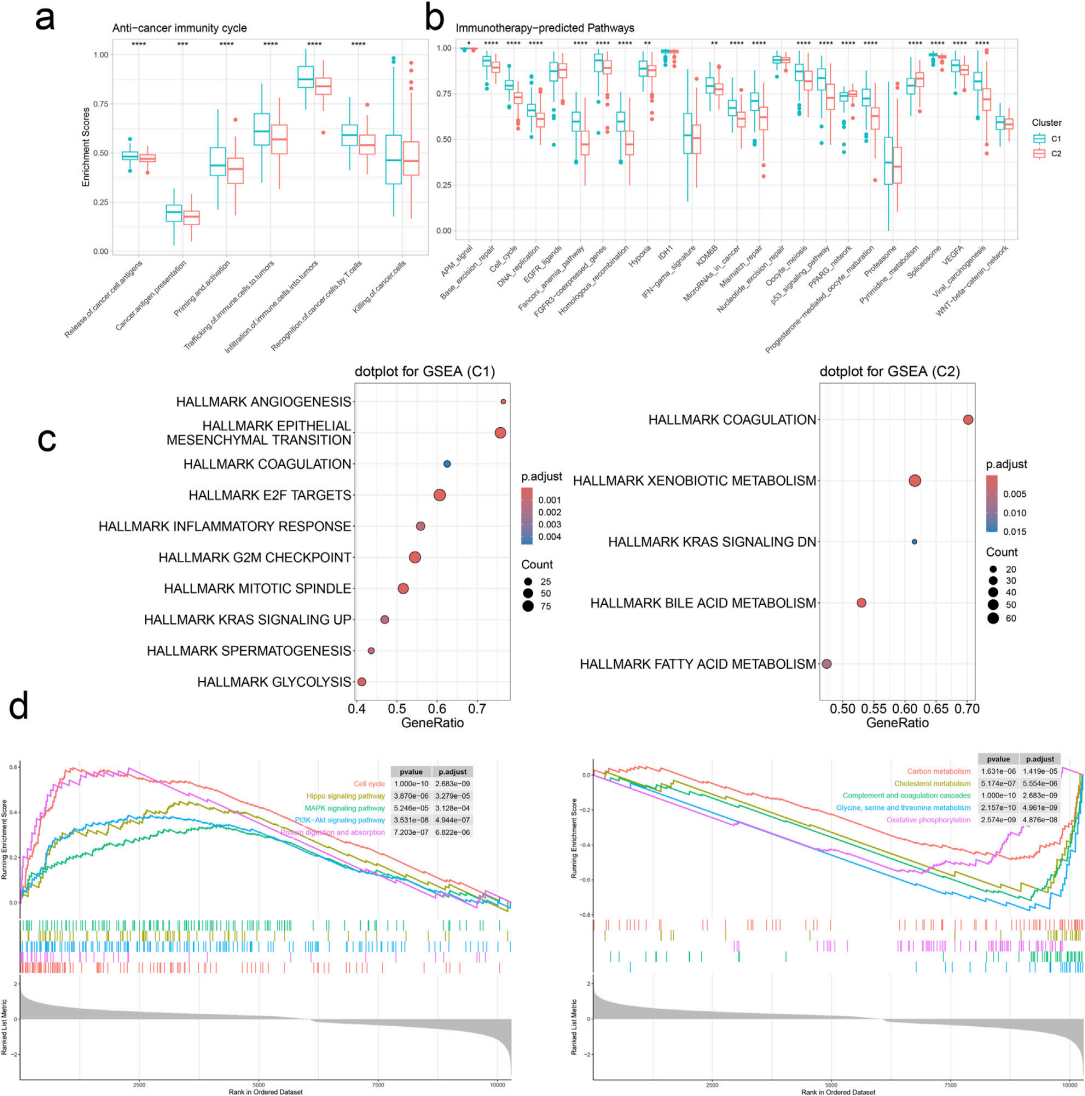
Signature stratifies HCC TME into two subclusters with distinct dysregulated pathways. **(A)** The activities of anti-cancer immunity between two clusters by GSVA. **(B)** The activities of immunotherapy-predicted pathways between two clusters by GSVA. *p < 0.05, **p < 0.01, ****p < 0.0001. **(C)** Upregulated cancer hallmarks in the two clusters by GSEA. **(D)** Upregulated (left panel) and downregulated (right panel) pathways in C1.

We commenced our investigation by analyzing the relative infiltration of immune cell subtypes within C1 and Cluster 2(C2). C1 exhibited higher relative infiltration rates in Activated CD4 T cells, Central memory CD4 T cells, Central memory CD8 T cells, and Effector memory CD4 T cells compared to C2, while C2 demonstrated higher relative infiltration rates in CD56bright natural killer cells, eosinophils, Regulatory T cells, and T follicular helper cells compared to C1 (Figure 5E). Moreover, C1 surpassed C2 in CYT indicators, indicating heightened cytotoxic activity within C1, which may confer a favorable anti-tumor response (Figure 5F). In the analysis of immune cell infiltration, C1 showed elevated levels compared to C2 in Activated CD4 T cells, Central memory CD4 T cells, Central memory CD8 T cells, Effector memory CD4 T cells, Regulatory T cells, T follicular helper cells, and Type 2 T helper cells, while C2 exhibited higher levels in CD56bright natural killer cells and eosinophils. Overall, C1 displayed higher infiltration levels compared to C2 in the MCPcounter, quanTlseq, EPIC, and TIMER analyses, except for the “Other” category, where C2 was higher (Figure 6A). Furthermore, in the immune modulator expression profile, we categorized 150 factors into 5 classes (chemokine, Immunoinhibitor, Immunostimulator, MHC, receptor). Notably, C1 and C2 exhibited significant disparities in immune infiltration, with C1 displaying markedly higher overall abundance than C2 (Figure 6B). In the anti-cancer immunity cycle, Enrichment Scores (ES) of C1 consistently exceeded those of C2, with the majority of pathways in the immunotherapy-predicted pathway graph favoring C1 (Figures 7A, B).

### 3.6 Gene set enrichment analysis enrichment analysis

We conducted Gene Set Enrichment Analysis (GSEA) to identify the pathways enriched in C1 and C2. Notably, C1 exhibited significant enrichment in HALLMARK EPITHELIAL-MESENCHYMAL TRANSITION, HALLMARK E2F TARGETS, and HALLMARK G2M CHECKPOINT, while C2 showed prominent enrichment in HALLMARK XENOBIOTIC METABOLISM. Additionally, we observed that C1 cluster harmoniously upregulated pathways related to Cell cycle, Hippo signaling pathway, MAPK signaling pathway, PI3K-Akt signaling pathway, and Protein digestion and absorption, while concurrently downregulating pathways associated with Carbon metabolism, Cholesterol metabolism, Complement and coagulation cascades, Glycine, serine, and threonine metabolism, and Oxidative phosphorylation (Figures 7C, D). Our findings are similar to those obtained from the differential gene enrichment pathway analysis of malignant tumor cell subpopulations and CAF-related subpopulations in single-cell sequencing. Our analytical results demonstrate certain enriched pathway characteristics of tumors from different data dimensions, indicating a degree of universality.

### 3.7 Construction and validation of prognostic model

**FIGURE 8:**
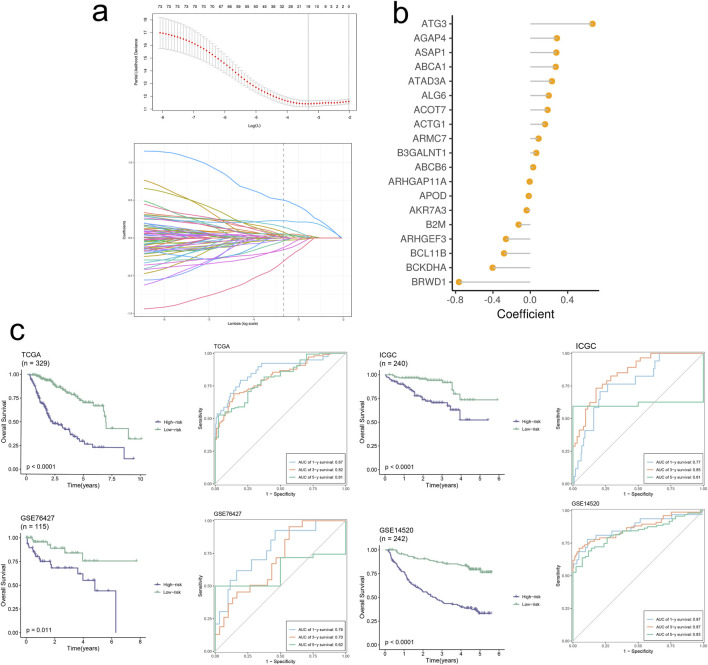
Signature-based model demonstrates high accuracy and robust performance in predicting prognosis. **(A)** The selection of prognostic signature genes based on the optimal parameter λ that was obtained in the LASSO regression analysis. **(B)** Lollipop chart of the coefficients of signature genes. **(C)** KM curves displayed survival outcomes of patients in two risk groups. Time-dependent ROC curves were drawn to assess survival rate at 1-year, 3-year, and 5-year.

**FIGURE 9:**
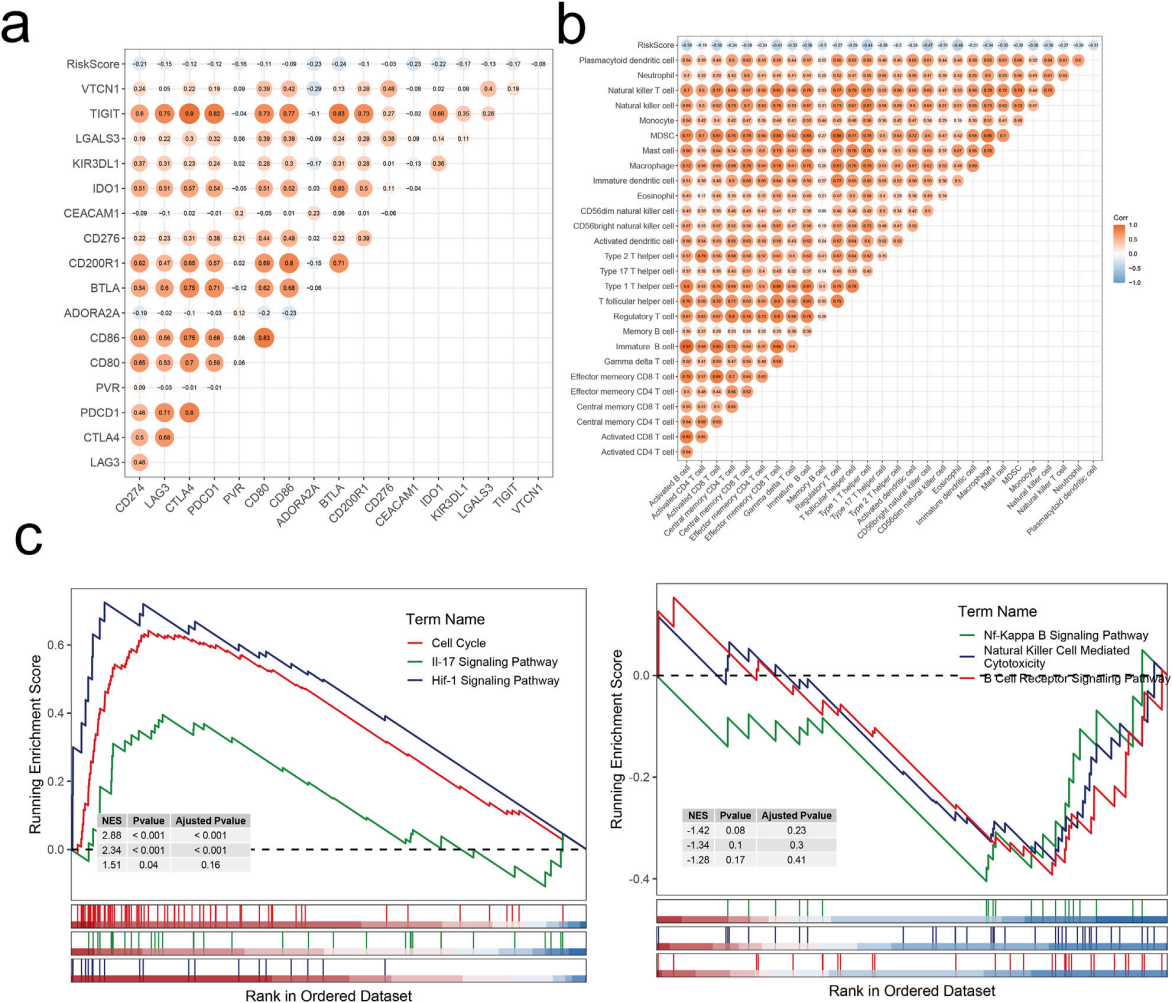
Correlation analysis and enrichment analysis. **(A)** Correlations between RiskScore and immune checkpoints. **(B)** Correlations between RiskScore and infiltration levels of 28 immune cell subsets. **(C)** Dysregulated pathways in high-risk LIHC patients.

The final set of 19 genes was obtained through stepwise Cox proportional hazards regression, with nonzero coefficients (Figures 8A, B). Subsequently, patients were scored using the model formula to derive individual RiskScores. Based on the median of RiskScore calculations, patients were stratified into high-risk and low-risk groups. As depicted in Figure 8C, patients in the high-risk group exhibited significantly poorer overall performance compared to those in the low-risk group across all four datasets (*p* < 0.01) according to the Kaplan-Meier curves. Our model demonstrated robust validation performance across the four datasets (AUC > 0.6). A multiple correlation analysis was conducted, revealing mostly negative correlations between Riskscore and model genes (Figure 9A). Additionally, Riskscore exhibited negative correlations with immune cell infiltration scores (Figure 9B). Differential expression gene analysis was performed using the “limma” package to compare high- and low-risk groups, followed by Gene Set Enrichment Analysis (GSEA) on the selected differentially expressed genes. Finally, GSEA was employed to identify pathways upregulated (three on the left) and downregulated (three on the right) in the high-risk group (Figure 9C). The above results are analogous to those obtained from the enrichment analysis of single-cell subpopulations.

### 3.8 ABCA1 plays a pro-oncogenic role in HCC cells

**FIGURE 10:**
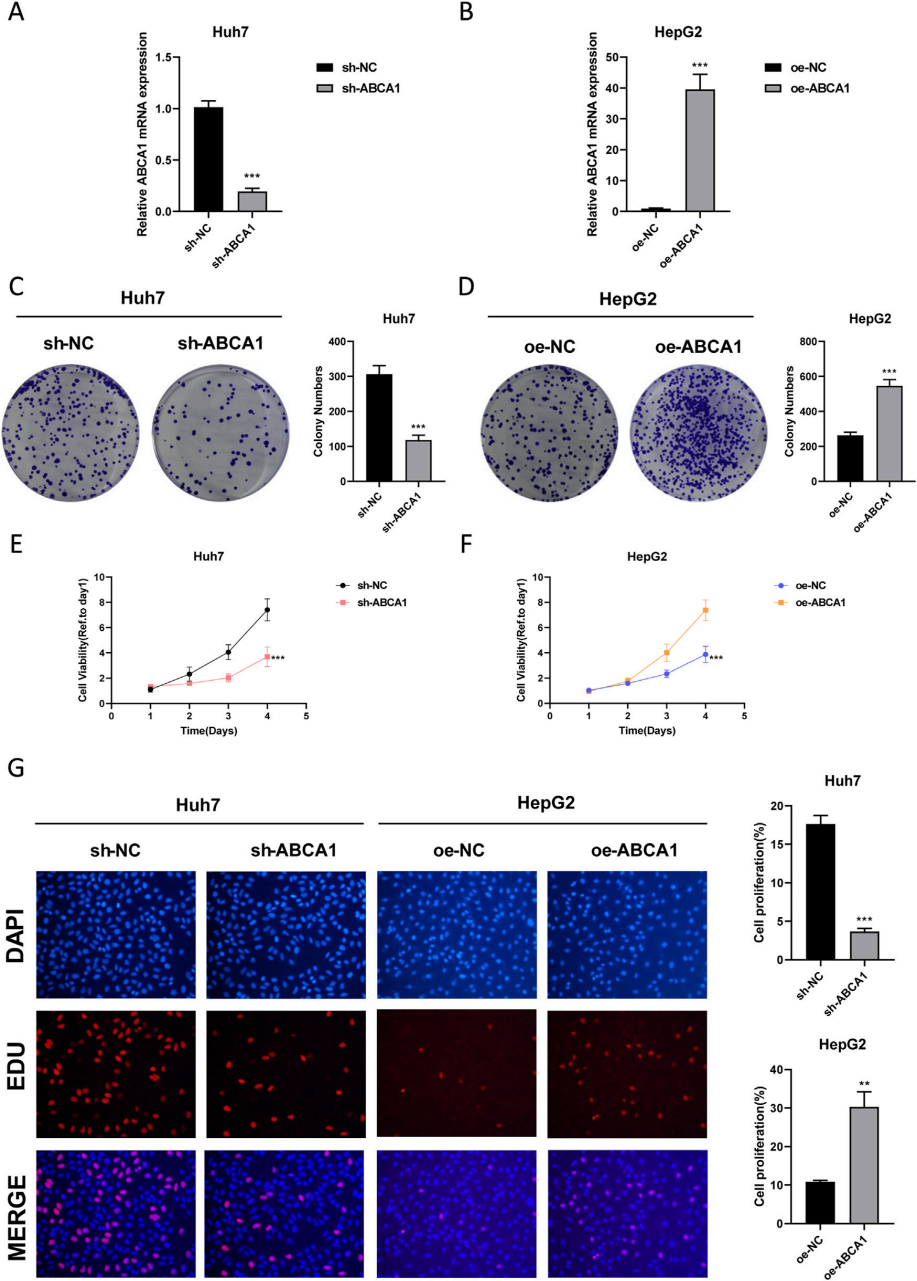
Efficiency validation of ABCA1 knockdown and overexpression and their impact on cancer cell proliferation. **(A)** RT-qPCR experiment validating the knockdown efficiency of sh-ABCA1 in Huh7 cell line. **(B)** RT-qPCR experiment validating the overexpression efficiency of oe-ABCA1 in HepG2 cell line. **(C)** Colony formation assay reflecting differences in proliferation levels between ABCA1 knockdown group and control group cells. **(D)** Colony formation assay reflecting differences in proliferation levels between ABCA1 overexpression group and control group cells. **(E)** CCK8 assay reflecting differences in proliferation levels between ABCA1 knockdown group and control group cells. **(F)** CCK8 assay reflecting differences in proliferation levels between ABCA1 overexpression group and control group cells. **(G)** EDU assay reflecting differences in proliferation levels between ABCA1 knockdown group, ABCA1 overexpression group, and control group cells.

**FIGURE 11:**
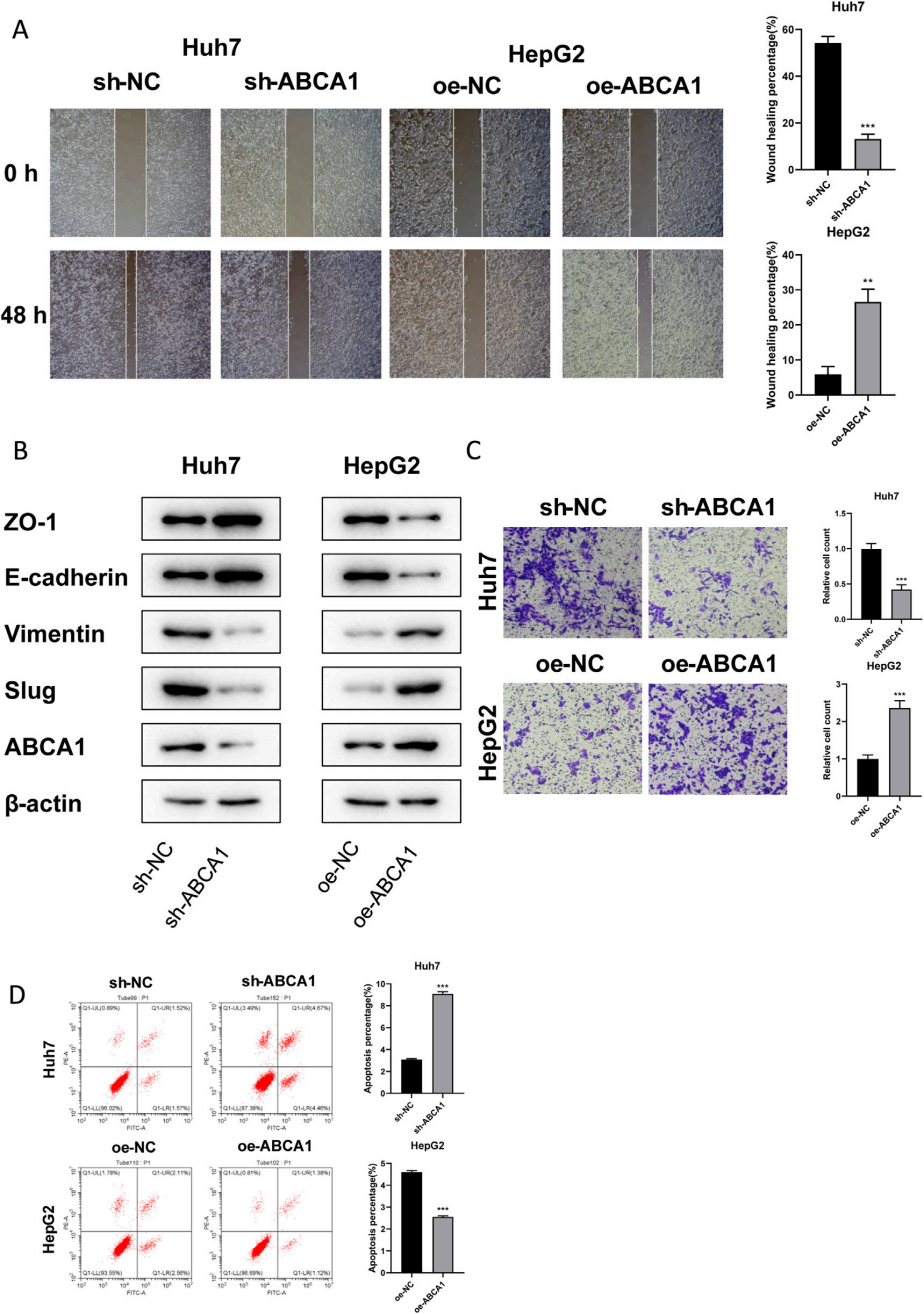
Effects of ABCA1 knockdown and overexpression on cell migration, invasion, and apoptosis capabilities. **(A)** Wound healing assay validating differences in migration levels between ABCA1 knockdown group, ABCA1 overexpression group, and control group cells. **(B)** Western blot validating differences in migration-related protein expression levels between ABCA1 knockdown group, ABCA1 overexpression group, and control group cells. **(C)** Transwell assay validating differences in invasion levels between ABCA1 knockdown group, ABCA1 overexpression group, and control group cells. **(D)** Flow cytometry validating differences in apoptosis levels between ABCA1 knockdown group, ABCA1 overexpression group, and control group cells.

RT-qPCR analysis revealed that knockdown of ABCA1 significantly reduced ABCA1 mRNA expression in Huh7 cells compared to controls. Conversely, overexpression of ABCA1 markedly increased ABCA1 mRNA expression in HepG2 cells (*p* < 0.001, Figures 10A, B). Colony formation assays demonstrated fewer colonies in the ABCA1 knockdown group of Huh7 cells and more colonies in the ABCA1-overexpressing group of HepG2 cells, indicating a role for ABCA1 in promoting HCC cell proliferation (*p* < 0.001, Figures 10C, D). CCK8 assays showed decreased cell viability in Huh7 cells following ABCA1 knockdown, whereas increased viability was observed in HepG2 cells upon ABCA1 overexpression (*p* < 0.001, Figures 10E, F). EDU assays indicated reduced proliferation in Huh7 cells with ABCA1 knockdown compared to controls (*p* < 0.001), and increased proliferation in HepG2 cells with ABCA1 overexpression (*p* < 0.01, Figure 10G). Wound healing assays demonstrated reduced cell migration capability following ABCA1 knockdown (*p* < 0.001), and enhanced migration upon ABCA1 overexpression in HepG2 cells (*p* < 0.01, Figure 11A). Western blot analysis revealed significant expression differences of ZO-1, E-cadherin, Vimentin, and Slug proteins between normal and overexpressing ABCA1 conditions in both Huh7 and HepG2 cells (Figure 11B). Transwell assays showed increased invasive cell counts in both control groups, with significantly higher invasion in cells expressing higher levels of ABCA1 (*p* < 0.001, Figure 11C). Flow cytometry analysis indicated a higher apoptotic percentage in cells with lower ABCA1 expression, suggesting a role for ABCA1 in reducing apoptosis in HCC cells (*p* < 0.001, Figure 11D). In summary, ABCA1 plays a pro-oncogenic role in HCC cells by promoting proliferation, invasion, migration, and reducing apoptosis.

## 4 Discussion

HCC represents a major histological subtype of liver cancer and ranks among the deadliest malignancies. According to relevant data, the global number of new HCC cases reached 905,677 in 2020, with 830,180 new deaths reported (Foerster et al., 2022). Despite advancements in therapeutic strategies, the mortality rate of HCC remains high, primarily due to its late-stage diagnosis. Once HCC progresses to an advanced stage, it becomes highly invasive with a dismal prognosis, resulting in a 5-year survival rate of around 20% for patients (Chen et al., 2022).

The optimal treatment for HCC is liver resection or transplantation, yet the surgical cure rate is only about 20%, and surgical indications are stringent, leaving most patients with conservative treatment options (Llovet et al., 2024). Concurrently, CAFs have been implicated in HCC’s tumor proliferation, angiogenesis, metastasis, and chemotherapy resistance (Biffi and Tuveson, 2021). Therefore, investigating the correlation between malignant cells in HCC and CAFs using bioinformatics techniques, analyzing the role of HCC-related genes and signaling pathways in the TME, and constructing prognostic models are of significant importance. Furthermore, the screening and analysis of differentially expressed genes contribute to early diagnosis and precision treatment of HCC.

We downloaded bulk transcriptomic data and corresponding clinical data of HCC from the public database TCGA, datasets GSE14520 and GSE76427 from the GEO database, ICGC-JP dataset from the ICGC database, and three single-cell sequencing datasets GSE146115, GSE146409, and GSE166635 from the TISCH2 database. These datasets hold immense research and application potential in the diagnosis, treatment, and prognostic assessment of patients.

After quality control, we performed UMAP dimensionality reduction on the single-cell sequencing data, resulting in 28 clusters, annotated into 12 major cell types. Further UMAP dimensionality reduction was conducted on 2 cell types—malignant cell clusters based on EPCAM expression levels and CAFs cell clusters based on COL1A1 and COL1A2 expression levels—yielding 6 malignant cell subgroups and 3 CAFs cell subgroups. By constructing developmental lineages and trajectories for each cell subgroup, we observed that each malignant cell subgroup generally exhibited two developmental trajectories, with the Malignant_Epi_0 cell subgroup likely being their common developmental origin. In contrast, each CAFs cell subgroup had only one developmental trajectory, with an unknown developmental origin. We conducted DEGs analysis for each cell subgroup. Among the 6 malignant cell subgroups, MT-ND3, MT-CYB, MT-ATP6, MT-CO2, and MT-CO1 were identified as differentially expressed genes in the Malignant_Epi_2, Malignant_Epi_3, and Malignant_Epi_4 cell subgroups, while ALB, AHSG, and APOE were also identified as differentially expressed genes in the Malignant_Epi_1 and Malignant_Epi_5 subgroups, exhibiting a consistent downregulation trend across all subgroups. We infer that the downregulation of these genes may promote tumor proliferation and metastasis, leading to unfavorable prognosis. Among the 3 CAFs cell subgroups, RPL17-C18orf32, AC135178.2, and MTRNR2L8 were identified as differentially downregulated genes in CAF_0 and CAF_2, but exhibited an upregulation trend in CAF_1. Finally, we conducted GO_BP enrichment analysis and extracted the top six statistically significant signaling pathways for each cell subgroup.

To delve deeper into the correlation between malignant cells and CAFs, we conducted cell communication analysis on various cell subpopulations using the CellChat and NicheNet algorithms. Beyond examining the frequency and strength of interactions between each cell subpopulation, we also investigated the receptor relationships of different pathways within these subpopulations. Specifically, we focused on the SPP1 signaling pathway to elucidate the ligand-receptor pairing status. Through detailed analysis of various components of the SPP1 signaling pathway, we observed that the Malignant_Epi_0 cell subpopulation is crucial in all four aspects—Sender, Receiver, Mediator, and Influencer—while the Malignant_Epi_3 subpopulation exhibits the opposite pattern, indicating divergent modes of action concerning SPP1. We also studied the outward and inward interaction strengths of each cell subpopulation. Finally, by pairing genes expressed by CAFs as ligands with genes expressed by malignant cells as receptors, we analyzed their binding potential and biological efficacy. We found that only when IL1B acts as the ligand and IL1RAP, IL1R1, and IL1R2 act as receptors, both binding potential and biological effects are evident. Furthermore, we constructed GRNs based on single-cell data from HCC. During this process, we focused on the distribution and expression of five regulatory factors associated with HCC across different cell subpopulations: BRF1_extended_29g, ARNTL_extended_39g, ARNTL_24g, BCLAF1_extended_22g, and ATF3_extended_16g. BRF1 encodes one of the three subunits of RNA polymerase Ⅲ transcription factor complex, which plays a core role in initiating transcription of genes encoding tRNA, 5S rRNA, and other small structural RNAs. Studies have shown that BRF1 is highly expressed in human tumor tissues of HCC patients, and inhibiting its expression can suppress HCC development (Lin et al., 2020). ARNTL encodes a protein with a basic helix-loop-helix structure and has been shown to exert anti-tumor effects in many human cancers. Downregulation of ARNTL in HCC patients promotes growth and metastasis of HCC cells both *in vitro* and *in vivo*, significantly correlating with low survival rates (Yang et al., 2022). BCLAF1 interacts with members of the Bcl2 family of anti-apoptotic proteins and enhances HIF1α expression in HCC tissues under hypoxic conditions, thereby promoting HCC-related angiogenesis and disease progression. Therefore, BCLAF1 is likely to be a therapeutic target for anti-proliferation and anti-angiogenesis treatment in HCC (Wen et al., 2019). ATF3, a member of the cAMP responsive element-binding protein (CREB) family, has been found to be a tumor suppressor that inhibits proliferation and metastasis of HCC cells. It also significantly correlates with intrahepatic metastasis and overall survival (OS) of HCC patients (Chen et al., 2018). For these five regulatory factors, we also explored differences in their activity levels between malignant and CAF cells. Subsequently, we merged the target genes of these five regulatory factors to obtain a signature for unsupervised clustering analysis.

We applied unsupervised clustering analysis to hierarchically cluster tumor tissue samples, aiming to categorize the samples. By selecting the most suitable value for (k) [where (k = 2)], we partitioned the samples into two distinct clusters, denoted as C1 and C2. Subsequently, we performed KM survival analysis on each cluster, which showed that the survival rates for both clusters decreased over time. Following this, we compared the TMEs of the two clusters.

First, we utilized ssGSEA to score 28 immune cell subsets to measure their relative infiltration abundance. Comparing the statistically significant data, we found that in cluster C1, there were higher levels of immune cell infiltration for Activated CD4 T cells, Effector Memory CD4 T cells, Regulatory T cells, and Type 2 T helper cells. Conversely, in cluster C2, Eosinophils exhibited higher levels of infiltration compared to cluster C1. These findings suggest that each cluster may play distinct and significant roles in different immune response regulations.

Next, we analyzed the expression levels of CYT, GFP, IFNG, and TMB between clusters C1 and C2, finding that only CYT showed statistically significant differences, with cluster C1 exhibiting significantly higher activity than cluster C2. To further understand the TMEs, we used five different algorithms—CIBERSORT, MCP-counter, quanTIseq, EPIC, and TIMER—to assess the infiltration levels of immune cell subsets in the two clusters. The results aligned with our earlier findings.

We then extracted data for 150 immunomodulators and chemokines from the TISIDB database, including chemokines, Immunoinhibitors, Immunostimulators, MHC, and receptors. We analyzed their expression patterns in both clusters. The results indicated that these five types of substances were generally highly expressed in cluster C1 and under-expressed in cluster C2. This could suggest that cluster C1 is more closely associated with immune regulation and immune response, while cluster C2 might be involved in the inhibition and regulation of immune activity.

Finally, we used GSVA to measure the enrichment scores for the anti-cancer immunity cycle and immunotherapy-predicted pathways in the two clusters. Upon observation, we noted that the C1 cluster exhibited higher Enrichment Scores in both the anti-cancer immunity cycle and immunotherapy-predicted pathway. Therefore, we reasonably infer that target genes within the C1 cluster play a pivotal role in the regulation and treatment of anti-cancer immunity. This finding contributes to a better understanding of the mechanisms underlying different cell clusters in immunotherapy, while also providing significant guidance for the formulation of cancer treatment strategies.

We utilized the “limma” package to identify differential genes between the C1 and C2 clusters and performed GSEA. This revealed upregulated cancer signatures in both clusters. Specifically, in the C1 cluster, upregulated cancer signatures were closely associated with various aspects of tumor initiation, progression, immune microenvironment, metastasis, and cell cycle regulation. Conversely, upregulated cancer signatures in the C2 cluster implicated multiple metabolic pathways, suggesting that modulating aberrant metabolic pathways might be a crucial therapeutic strategy in HCC treatment. Additionally, GSEA helped identify upregulated and downregulated signaling pathways in the C1 cluster. Analysis revealed that upregulated signaling pathways were linked to tumor cell proliferation and signal transduction, while downregulated pathways involved fundamental metabolic processes such as the complement and coagulation cascade, energy metabolism, and protein synthesis. Overall, the abnormal proliferation of cells in the C1 cluster, coupled with suppressed metabolic processes, exacerbates tumor growth, dissemination, and metastasis. Furthermore, the downregulation of the complement and coagulation cascade pathway may be associated with the abnormal coagulation status observed in HCC patients.

Utilizing the TCGA-LIHC dataset, we employed LASSO and multiCOX analysis methods to construct a HCC prognostic model and assigned scores to model factors, yielding a RiskScore for each sample. Based on the median score, we stratified samples into high and low-risk groups. Subsequently, KM curves were plotted to predict prognosis for both high and low-risk groups, revealing a progressive decrease in survival rates over time for both groups, with notably poorer prognosis observed in the high-risk group. We assessed the model’s diagnostic performance at 1, 3, and 5-year time points through ROC curve analysis, demonstrating good performance. Furthermore, we validated the prognostic model in three external datasets (GSE76427, GSE14520, ICGC-JP) using KM and ROC curve analyses, showing excellent accuracy and predictive ability across different datasets.

We then examined the correlation between RiskScore and various immune checkpoint levels and immune cell infiltration levels. Differential gene expression analysis was performed to identify DEGs between high and low-risk groups. Subsequently, GSEA revealed dysregulated signaling pathways in high-risk group patients. Analysis indicated that upregulated signaling pathways in the high-risk group were associated with tumor cell proliferation and cell cycle regulation, promoting malignant tumor growth and development. Conversely, downregulated signaling pathways were linked to anti-tumor immune responses and immune regulation, likely facilitating tumor immune evasion and affecting the regulation of the TME, thus exerting significant adverse effects on HCC prognosis. These inferences also corroborated the accuracy of our prognostic model.

Single-cell sequencing, with its outstanding resolution, demonstrates significant advantages over bulk sequencing in elucidating disease mechanisms. However, considering cost-effectiveness and the convenience of large-scale application, bulk sequencing still holds its ground. Therefore, combining these two technologies for comparative analysis can fully leverage their respective strengths. In this study, we conducted in-depth differential expression and enrichment analyses on malignant cell subpopulations and CAF subpopulations at the single-cell level, while exploring differential gene enrichment among different clusters and model risk groups at the bulk sequencing level.

The comparative analysis revealed that both single-cell sequencing and bulk sequencing identified the universal upregulation of metabolic pathways in the tumor microenvironment, suggesting that metabolic reprogramming may be a common feature in tumor development. Furthermore, both technologies observed the activation of cell signaling transduction-related pathways, which are closely related to the proliferation and migration of tumor cells. Notably, single-cell sequencing uniquely captured the upregulation of immune inflammation and neuroregulatory-related pathways in malignant cell subpopulations, which were not explicitly identified in bulk sequencing, highlighting the powerful ability of single-cell sequencing in resolving cell subpopulation-specific characteristics. On the other hand, bulk sequencing detected the upregulation of pathways related to epithelial-mesenchymal transition (EMT), a finding not directly reflected in single-cell sequencing. Given that EMT is a complex process involving multiple cell subpopulations and pathway interactions, it may be implicitly manifested in single-cell sequencing as differential expression patterns among different subpopulations. Furthermore, we speculate that the upregulation of metabolic and signal transduction pathways observed in single-cell sequencing may be intrinsically linked to the activation of cell cycle regulation, Hippo signaling pathway, MAPK signaling pathway, and PI3K-Akt signaling pathway observed in bulk sequencing, all of which jointly contribute to the proliferation and survival of tumor cells.

Despite the differences in pathway analysis between single-cell sequencing and bulk sequencing, they both emphasize the complexity and heterogeneity of the tumor microenvironment. This heterogeneity may arise from interactions among different cell subpopulations and the diversity of pathway regulation. By integrating the results of these two sequencing technologies, we hope to gain a deeper understanding of the molecular mechanisms underlying tumor development and progression, and provide new perspectives and ideas for the formulation of future therapeutic strategies.

We conducted knockdown and overexpression experiments of ABCA1 in two HCC cell lines. Subsequent phenotypic assays confirmed that ABCA1 exerts a pro-oncogenic effect in HCC cells by promoting proliferation, invasion, migration, and reducing apoptosis. Our wet lab experiments corroborate the bioinformatic findings, providing robust evidence for the role of ABCA1 in liver cancer. This study not only reinforces the computational results but also lays a foundation for future research.

However, our study still has certain limitations. We are acutely aware that relying solely on *in vitro* experimental results poses significant constraints when directly translating to clinical applications. To bridge the gap in clinical translation, we plan to initially utilize animal models, particularly patient-derived xenograft (PDX) models and humanized mouse models that closely mimic the tumor characteristics of patients, to simulate a more authentic *in vivo* environment and further explore the functions and mechanisms of ABCA1. This will include, but is not limited to, assessing the specific effects of ABCA1 on tumor growth, metastasis, and the tumor immune microenvironment *in vivo*. Subsequently, we will employ high-throughput screening and precision medicine strategies to identify potential therapeutic targets for ABCA1 and develop corresponding therapeutic interventions. Furthermore, we will closely monitor changes in relevant biomarkers, with the aim of establishing a biomarker system that can predict treatment efficacy and patient prognosis. Our objective is to build a solid evidence base through advanced preclinical research to guide future clinical trials and facilitate the clinical translation of ABCA1-related research.

## 5 Conclusion

Through comprehensive integration of TCGA, GEO, ICGC, and TISCH2 databases, we conducted single-cell sequencing analysis and cell communication analysis on multiple malignant and CAFs cell subpopulations, revealing the functional characteristics and receptor relationships of each cell subgroup. Additionally, we constructed GRNs, delving into the regulatory factors associated with HCC and their target genes. Utilizing an unsupervised clustering analysis based on target genes, we identified two clusters, C1 and C2, and analyzed their TME differences. Furthermore, through GSEA, we identified upregulated cancer features in two clusters and signaling pathways that were both upregulated and downregulated in the C1 cluster.

We constructed a prognostic model and assigned scores, grouping patients based on RiskScore and predicting their prognosis accordingly. The results demonstrated the excellent accuracy and clinical utility of our model. Additionally, we discovered a correlation between RiskScore, immune checkpoint expression, and immune cell infiltration levels. GSEA analysis revealed dysregulated signaling pathways in the high-risk group, adversely affecting HCC prognosis. Our study provides important insights for the prognostic evaluation and formulation of treatment strategies for HCC.

## Data Availability

The raw data supporting the conclusions of this article will be made available by the authors, without undue reservation.
